# Investigating Risk Factors for Complications after Ileostomy Reversal in Low Anterior Rectal Resection Patients: An Observational Study

**DOI:** 10.3390/jcm8101567

**Published:** 2019-10-01

**Authors:** Mateusz Rubinkiewicz, Jan Witowski, Michał Wysocki, Magdalena Pisarska, Stanisław Kłęk, Andrzej Budzyński, Michał Pędziwiatr

**Affiliations:** 1 Department of General Surgery, Jagiellonian University Medical College, Kopernika 21, 31-501 Krakow, Poland; mateusz.rubinkiewicz@uj.edu.pl (M.R.); jwitos@gmail.com (J.W.); michal92wysocki@gmail.com (M.W.); magdalenapisarska@interia.pl (M.P.); andrzej.s.budzynski@gmail.com (A.B.); 2 General and Oncology Surgery Unit, Stanley Dudrick’s Memorial Hospital, 32-050 Skawina, Poland; klek@poczta.onet.pl

**Keywords:** ileostomy, risk factors, digestive system surgical procedures, length of stay, postoperative period

## Abstract

**Introduction:** Defunctioning ileostomy has been widely used in patients undergoing low anterior rectal resection to reduce the rate of postoperative leakage. It is still not clear whether interval between primary procedure and ileostomy reversal has an impact on treatment outcomes. **Methods:** In our prospective observational study we reviewed 164 consecutive cases of patients who underwent total mesorectal excision with primary anastomosis. Univariate and multivariate regression models were used to search for risk factors for prolonged length of stay and complications after defunctioning ileostomy reversal. Receiver operating characteristic curves were utilized to set cut-off points for prolonged length of stay and perioperative morbidity. **Results:** In total, 132 patients were included in the statistical analysis. The median interval between primary procedure and defunctioning ileostomy reversal was 134 (range: 17–754) days, while median length of stay was 5 days (4–6 interquartile range (IQR)). Prolonged length of stay cut-off was established at 6 days. Regression models revealed that interval between primary surgery and stoma closure as well as complications after primary procedure are risk factors for complications after defunctioning ileostomy reversal. Prolonged length of stay has been found to be related primarily to interval between primary surgery and stoma closure. **Conclusions:** In our study interval between primary surgery and stoma closure along with complication occurrence after primary procedure are risk factors for perioperative morbidity and prolonged length of stay (LOS) after ileostomy reversal. The effort should be made to minimize the interval to ileostomy reversal. However, randomized studies are necessary to avoid the bias which appears in this observational study and confirm our findings.

## 1. Introduction

Rectal cancer affects 125,000 patients within the European Union every year, representing 44% of all colorectal cancer cases [1,2]. Surgical resection remains the gold standard, providing good long-term oncological outcomes [3,4]. Minimally invasive techniques have spread in rectal cancer surgery as well as in other branches in abdominal surgery [5,6,7]. It has been proven that the laparoscopic approach improves short-term outcomes without comprising oncological results [8,9]. Despite improvements in surgical technique and perioperative care, the number of postoperative complications remains high. Among them, anastomotic leakage significantly contributes to inferior oncological outcomes and higher mortality rates [10]. It has been shown that the incidence of anastomotic leakage is strongly correlated with these issues. In addition, healing of anastomosis may be compromised due to neoadjuvant radiochemotherapy [11]. Therefore, defunctioning ileostomy has been widely used in patients undergoing low anterior rectal resection to reduce the rate of postoperative leakage [12,13]. The optimal interval between the primary procedure and ileostomy reversal has not yet been clearly established. The biggest drawback of the ileostomy approach is that, as with all surgical procedures, it may be associated with perioperative complications and compromised recovery, leading to prolonged length of hospital stay (LOS) and delay in adjuvant treatment. For this reason, the identification of factors contributing to postoperative morbidity and prolonged LOS seems to be of great clinical importance.

The aim of this study was to identify the risk factors for postoperative complications and prolonged hospital stay after ileostomy reversal in patients after low anterior rectal resection for cancer.

## 2. Materials and Methods

This was a prospective observational study with post-hoc analysis of patients who underwent ileostomy reversal procedure after rectal resection due to malignancy in two high-volume hospitals. To identify the risk factors of postoperative complications and prolonged hospitalisation after ileostomy reversal, a patient database was created. The inclusion criteria were written informed consent on hospital admission, and total mesorectal excision with defunctioning ileostomy. Every patient was treated according to the Enhanced Recovery After Surgery (ERAS) protocol [14,15]. Every patient had endoscopic examination after surgery. If the anastomosis was healed, the patients were instructed to start an anal sphincter exercise programme with follow-up visits in the outpatient department. After achieving satisfactory results of faecal continence, the ileostomy reversal procedure was scheduled, either before the start of adjuvant treatment (approximately 21 days after initial procedure) or 30 days after termination of adjuvant chemotherapy. If after initial procedure patient required prolonged hospitalisation (due to perioperative complications), delayed ileostomy reversal was scheduled.

**Figure 1:**
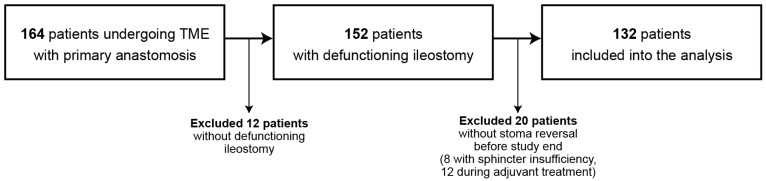
Flowchart of patients included in study with reasons for exclusion from the analysis. TME: total mesorectal excision.

A retrospective review of 164 consecutive rectal cancer patients who underwent total mesorectal excision followed by primary anastomosis was done (Figure 1). Twelve patients did not have a defunctioning ileostomy created. Out of remaining 152 patients, 20 did not have a stoma reversal during study duration (8 patients had complete sphincter insufficiency and 12 were in adjuvant treatment). Finally, 132 patients who underwent ileostomy reversal were included in the analysis.

The primary endpoint of the study was risk factors identification for postoperative morbidity and length hospitalisation after defunctioning ileostomy reversal. The secondary endpoint was risk factors identification for prolonged hospitalisation after ileostomy reversal.

The LOS was calculated from the day of hospital admission (one day before defunctioning ileostomy reversal) to final discharge from hospital. Prolonged hospitalisation was considered as LOS longer than 75th percentile of all LOS. Patients were discharged regardless of the day of the week. Every patient also had an outpatient follow-up appointment scheduled 7 days after discharge. Postoperative complications were assessed according to the Clavien–Dindo classification [16].

### Statistical Analysis and Ethical Consideration

All data were analysed with Statistica version 13.0 PL (StatSoft Inc., Tulsa, OK, USA). The results are presented as mean standard deviation (SD), median, and interquartile range (IQR). The study of categorical variables used the chi-square test of independence. The Shapiro–Wilk test was used to check for normal distribution of data, and the Student’s *t*-test was used for normally distributed quantitative data. For non-normally distributed quantitative variables, the Mann-Whitney *U* test was used. A *p*-value <0.05 was considered statistically significant. All considerable patient- and treatment-related factors were analysed in univariate logistic regression models, then significant factors were analysed in the multiple logistic regression model in search of independent risk factors for prolonged LOS. Receiver operating characteristic (ROC) curves were used for setting cut-off points separately for prolonged LOS and perioperative morbidity.

All procedures were performed in accordance with the ethical standards of the 1964 Declaration of Helsinki and its later amendments (Fortaleza 2013). The study was approved by the Local Ethics Committee of the Jagiellonian University Medical College. Every patient signed an informed consent prior to inclusion in the study.

## 3. Results

Overall 132 patients were included in the study. Ninety-seven patients (73%) required adjuvant chemotherapy. Median interval from primary procedure to defunctioning ileostomy reversal was 134 (IQR 53–230, range 17–754) days. Median LOS calculated in the study group was 5 days (4–6 IQR). Prolonged hospitalisation cut off was calculated as 6 days. 72 (55%) patients had end to end anastomosis and 60 (45%) had side to side anastomosis. Twenty patients (15%) had postoperative complications, including 12 patients with surgical site infection (SSI). There were no cases of anastomotic leakage after ileostomy reversal. No reoperations were required within 30-day postoperative period. No mortality was noted in the study group. Twenty-seven patients (20 %) required prolonged hospitalisation. The full characteristics of the group are presented in Table 1 and Table 2. Perioperative complications are presented in Table 3. No complications after ileostomy reversal regarding primary anastomosis, such as abscess or stricture were noted.

Both univariate and multivariate logistic regressions showed that interval between primary surgery and stoma closure (univariate: odds ratio (OR) 4.39, 95% confidence interval (CI): 1.61–11.96, *p* = 0.003; multivariate: OR 7.17, 95% CI: 1.79–28.73, *p* = 0.005) and complications after primary procedure (univariate: OR 7.17, 95% CI: 1.79–28.73, *p* = 0.005; multivariate: OR 5.00, 95% CI: 1.21–20.77, *p* = 0.024) are risk factors for perioperative complications after defunctioning ileostomy reversal (Table 4).We also calculated risk factors for prolonged hospitalisation after ileostomy reversal. Univariate logistic regression analysis identified that anastomotic leak after primary procedure (OR 6.59; 95% CI: 10.08–40.28, *p* = 0.038), interval between primary surgery and stoma closure (OR 7.5; 95% CI: 2.86–19.66, *p* = 0.001), and any complication after stoma closure (OR 17.77; 95% CI: 5.75–54.90, *p* = 0.001) were related to prolonged LOS.

Next, the multivariate logistic regression identified that only interval between primary surgery and stoma closure (OR 18.39; 95% CI: 3.57–94.70, *p* = 0.001) is an independent risk factor for prolonged LOS after ileostomy reversal (Table 5).

## 4. Discussion

In our study we presented that the interval between primary procedure and ileostomy reversal and postoperative morbidity after primary procedure are independent risk factors of postoperative complications after ileostomy reversal in patients after low anterior rectal resection due to cancer. Regarding prolonged length of stay, we identified the interval between the primary procedure and ileostomy reversal as independent risk factor.

There is no doubt that defunctioning ileostomy is beneficial for the patients as it reduces the rate of anastomotic leakage after TME [17,18]. Also, patients with defunctioning ileostomy have lower risk of reoperation [17]. Moreover, it allows the use of anastomosis rescue techniques in the case of leakage, for example by application of EndoVac^®^ treatment. Also, the interval between the primary procedure may be an opportunity for the patients with poor sphincter function for biofeedback training; the results are unequivocal [19,20]. A study concerning this issue by Kim et al. was even terminated early due to no benefit of this technique [20]. Apart from all advantages of defunctioning ileostomy, there are also negative features. Firstly, defunctioning ileostomy may increase the ratio of overall morbidity [17]. It was also identified as a risk factor for faecal incontinence after ileostomy reversal [21,22,23]. It is also worth mentioning that ileostomy diminishes patient quality of life [22]. Moreover, it is important that some patients also end up with a definitive stoma due to comorbidities, or they may choose not to undergo a second procedure [24]. The risk of a definitive stoma may reach up to 22% [24]. Also, an ileostomy is much more difficult to care for than a colostomy, so the latter should be considered in patients with high risk of permanent stoma [25]. However, the evidence supports defunctioning ileostomy use, although efforts to limit the indications to selected cases might be considered. Moreover, alternative methods might be used to avoid ileostomy creation. Some authors advocate a technique called ghost ileostomy, in which the small bowel loop is exteriorized and, in case of the anastomotic leakage, the ileostomy is opened, becoming a fully functional ostomy [26,27]. In case of no evidence of complications, the bowel loop is repositioned into the peritoneal cavity; thus no additional anastomosis is needed [26,27]. In our unit we create defunctioning ileostomy routinely, since most of our patients undergo neoadjuvant chemoradiotherapy. The resignation from ileostomy creation is reserved for selected cases only.

Despite the range of the problem, the optimal moment for defunctioning ileostomy reversal has not been established. As ileostomy diminishes quality of life, patients are willing to undergo early ileostomy reversal. However, many factors may postpone the reversal procedure. Next, complications of the primary procedure require extensive treatment; therefore, they extend the interval to ileostomy reversal. Moreover, there are patients, who require anal sphincter rehabilitation due to poor faecal continence. The abovementioned groups of patients are candidates for delayed ileostomy reversal. Furthermore, a large percentage of patients require adjuvant chemotherapy administration, which sometimes delays ileostomy reversal until the end of treatment due to fear of postoperative complications after stoma closure [28]. However, in our material 73% of patients required adjuvant treatment, meaning that one of four did not get any treatment after surgery and early stoma closure would ideally fit to their treatment plan. Moreover, our patients developed only Clavien–Dindo I and II grades of complications, which are mild and would not be associated with adjuvant treatment delay. Therefore, reversal before the start of chemotherapy should be considered. According to an randomized control trial (RCT) by Kłęk et al. that analysed early closure, the start of adjuvant treatment was delayed by approximately 5 days in early closure group, which does not have influence on further treatment [29]. Nonetheless, an interesting fact was revealed in our study—postoperative morbidity after primary procedure was a risk factor for postoperative complications after ileostomy reversal. This gives rise to a discussion over whether this group of patients should be candidates for delayed reversal.

In our study, the interval between primary procedure and ileostomy reversal was found to be a risk factor for postoperative complications and prolonged LOS. It seems that earlier closure is beneficial, with the issue being the definition of “early”. Zhou considered a 90-day interval as early closure and proved its safety, even during adjuvant treatment [30]. Farag performed a meta-analysis of randomized controlled trials, and recommended a 14-day interval between the primary procedure and ileostomy reversal with comparable risk of perioperative complications with delayed reversal [28]. Kłęk et al. proved also that a 14-day interval is safe and moreover cost-effective, since it decreases the costs of stoma care equipment tenfold [29]. Early reversal is also associated with easier abdominal wall closure [31]. Danielsen et al. proved in a randomized, multicentre trial that very early stoma reversal (8–13 days after initial procedure), is also safe and feasible in patients with no radiological evidence of anastomotic leak [32]. Therefore, efforts should be made to increase the rate of early ileostomy closure as it is a safe, cost-effective procedure which improves patient quality of life.

Complications are well established independent risk factors for prolonged hospitalisation, and surgical site infection is the most common among them. To reduce its rate, Hsieh et al. recommend using purse-string closure of the skin after stoma reversal, which results in a lower infection rate and better cosmetic results [33]. Another idea to solve this problem is routine application of negative pressure wound treatment (NPWT), which was effective in abdominal surgery wound closure [34]. However, its efficacy was not confirmed in an RCT by Uchino et al., who investigated NPWT directly in stoma reversal procedures [35]. In our study, 12 patients overall had surgical site infection. In the case of SSI, all patients had NPWT treatment applied, which was effective in all cases. Five patients suffered from prolonged postoperative ileus. In all cases we used conservative treatment, which was effective. No reoperations were required after ileostomy reversal. All our patients who were submitted for ileostomy reversal had this procedure successfully done.

Our study has some limitations. Firstly, it is a retrospective study. Next, our patients were not randomly assigned to groups with early or delayed ileostomy reversal, which creates obvious bias. However, we used the multivariate analysis model to compensate for confounding variables. Furthermore, the study consisted of two surgical centres, which may limit future generalizations of the results, especially in terms of the approach for patient treatment. However, both of the centres that participated in the study are leading units in the ERAS protocol, and thus most of the principles are similar. Nonetheless, the technique of creating anastomosis was not standardized. We used the side-to-side technique when the distal bowel loop was narrow; however, the ultimate decision was up to the surgeon performing the ileostomy reversal. Also, we did not monitor the interval between adjuvant therapy completion and ileostomy reversal. In fact, the factor which delays reversal is postoperative chemotherapy, as reversal cannot be performed during that time. In this study we did not assess functional outcomes after ileostomy reversal. We explored this topic in our previous research, which revealed that defunctioning ileostomy was a risk factor of low anterior resection syndrome (LARS) in patients undergoing low anterior rectal resection [21]. Moreover, the prevalence of LARS syndrome in the abovementioned is still high and requires further investigation to limit its occurrence [36].

## 5. Conclusions

In our study, the interval between primary surgery and stoma closure, along with complication occurrence after the primary procedure, are risk factors for perioperative morbidity and prolonged LOS after ileostomy reversal. Efforts should be made to minimize the interval to ileostomy reversal. However, randomized studies are necessary to avoid the bias which appears in this observational study and confirm our findings.

## Figures and Tables

**Table 1:** Basic characteristics of the patients regarding primary procedure.

*n* (%)		132 (100%)
Males/females, *n* (%)		82/50 (62%/38%)
Mean age, years ± SD		59 ± 12
Mean BMI, kg/m^2^ ± SD		26.64 ± 4.76
Respiratory comorbidities, *n* (%)		5 (4%)
Cardiovascular comorbidities, *n* (%)		65 (49%)
Diabetes mellitus, *n* (%)		18 (14%)
ASA class	1	7 (5%)
	2	98 (74%)
	3	27 (21%)
Median WHO class (%)		2 (1–3)
WHO class	4	2 (1%)
	3	44 (32%)
	2	53 (39%)
	1	22 (17%)
	0	14 (11%)
Relaparotomy after primary procedure, *n* (%)		12 (9%)
Anastomotic leak after primary procedure, *n* (%)		10 (8%)
Postoperative chemotherapy, *n* (%)		97 (73%)
Any complication after primary procedure, *n* (%)		53 (40%)

ASA: American Society of Anestesiology; BMI: body mass index.

**Table 2:** Basic data of the patients before ileostomy reversal.

Median interval between primary procedure and stoma closure, days (IQR)	134 (53–230)
End to end anastomosis vs. side to side, *n* (%)	72/60 (55%/45%)
Median operative time of ileostomy closure, min (IQR)	85 (60–105)
First bowel movement, median POD (IQR)	2 (1–3)
Narrow distal loop of the ileostomy, *n* (%)	8 (6%)
Parastomal hernia, *n* (%)	22 (17%)

IQR: interquartile range; POD: postoperative day.

**Table 3:** Postoperative morbidity after ileostomy reversal.

Clavien–Dindo Grade	Type of Complication	*n* (%)
V	N/A	0
IV	N/A	0
IIIb	N/A	0
IIIa	N/A	0
II	Surgical site infection (NPWT treatment) Prolonged postoperative ileus	12 (9%) 5 (4%)
I	Wound hematoma	3 (2%)
Total		20 (15%)

NPWT: negative pressure wound treatment.

**Table 4:** Univariate and multivariate logistic regression models analyzing factors potentially influencing perioperative complications of stoma closure.

Factor	OR	95% CI	*p*-Value
**Univariate**			
Males/females	0.86	0.32–2.36	0.773
Age	1.04	0.99–1.08	0.086
BMI	1.12	0.96–1.30	0.143
Metabolic comorbidities	0.39	0.08–1.97	0.246
Cardiovascular comorbidities	0.59	0.17–2.03	0.393
Diabetes mellitus	0.59	0.17–2.03	0.393
Musculoskeletal comorbidities	0.83	0.09–7.84	0.871
ASA class	0.25	0.06–1.08	0.060
Number of routinely taken medications	0.79	0.58–1.08	0.140
Neoadjuvant chemoradiotherapy	0.90	0.26–3.13	0.863
WHO class	1.23	0.69–2.19	0.481
Postoperative chemotherapy	0.92	0.28–3.34	0.702
Operative time of primary procedure	0.99	0.98–1.04	0.119
End to end anastomosis vs. side to side	1.5	0.44–5.06	0.507
Operative time of ileostomy closure	0.98	0.96–1.01	0.062
First bowel movement	0.99	0.44–2.25	0.986
Narrow stoma outlet	0.13	0.005–302.14	0.610
Interval between primary procedure and stoma closure in days	4.39	1.61–11.96	0.003
Any complication after primary procedure	4.21	1.14–15.47	0.028
Peristomal hernia	2.81	0.70–11.34	0.139
Relaparotomy after primary procedure	4.65	0.88–24.59	0.066
Anastomotic leak after primary procedure	2.82	0.45–17.81	0.263
**Multivariate**			
Interval between primary procedure and stoma closure in days	7.17	1.79–28.73	0.005
Any complication after primary procedure	5.00	1.21–20.77	0.024

OR: Odds ratio; CI: confidence interval.

**Table 5:** Univariate and multivariate logistic regression models analysing factors potentially influencing LOS.

Factor	OR	95% CI	*p*-Value
**Univariate**			
Males/females	0.78	0.32–1.92	0.586
Age	1.04	0.99–1.08	0.057
BMI	1.04	0.92–1.18	0.496
Respiratory comorbidities	0.07	0.0001–233.32	0.654
Cardiovascular comorbidities	1.53	0.55–4.26	0.407
Diabetes mellitus	0.57	0.11–2.98	0.501
Musculoskeletal comorbidities	1.12	0.19–6.42	0.901
ASA class	1.14	0.41–3.21	0.795
Number of routinely taken medications	1.01	0.82–1.23	0.955
Neoadjuvant chemoradiotherapy	2.23	0.70–7.08	0.167
WHO class	1.26	0.76–2.07	0.366
Postoperative chemotherapy	0.43	0.18–1.06	0.065
Operative time of primary procedure	0.99	0.99–1.02	0.177
End to end anastomosis vs. side to side	0.66	0.23–1.87	0.428
Operative time of ileostomy closure	0.99	0.97–1.03	0.133
First bowel movement	0.82	0.40–1.67	0.576
Narrow stoma outlet	4.67	0.70–31.08	0.106
Interval between primary procedure and stoma closure in days	7.5	2.86–19.66	<0.001
Any complication after primary procedure	2.53	0.90–7.14	0.074
Parastomal hernia	1.95	0.55–6.96	0.294
Relaparotomy after primary procedure	4.31	0.86–21.76	0.072
Anastomotic leak after primary procedure	6.59	1.08–40.28	0.038
SSI after primary procedure	2.49	0.59–10.60	0.208
Wound hematoma	2.85	0.16–49.93	0.466
**Multivariate**			
Interval between primary procedure and stoma closure in days	18.39	3.57–94.70	<0.001
Anastomotic leak after primary procedure	6.29	0.59–67.57	0.122
